# Recent Challenges in Diagnosis and Treatment of Invasive Candidiasis in Neonates

**DOI:** 10.3390/children11101207

**Published:** 2024-09-30

**Authors:** Maria Baltogianni, Vasileios Giapros, Niki Dermitzaki

**Affiliations:** Neonatal Intensive Care Unit, School of Medicine, University of Ioannina, 45500 Ioannina, Greece; mbalt@doctors.org.uk (M.B.); n.dermitzaki@uoi.gr (N.D.)

**Keywords:** invasive candida infections, neonatal candidiasis, candida diagnosis, antifungal treatment

## Abstract

Invasive *Candida* infections represent a significant cause of morbidity and mortality in the neonatal intensive care unit (NICU), particularly among preterm and low birth weight neonates. The nonspecific clinical presentation of invasive candidiasis, resembling that of bacterial sepsis with multiorgan involvement, makes the diagnosis challenging. Given the atypical clinical presentation and the potential detrimental effects of delayed treatment, empirical treatment is often initiated in cases with high clinical suspicion. This underscores the need to develop alternative laboratory methods other than cultures, which are known to have low sensitivity and a prolonged detection time, to optimize therapeutic strategies. Serum biomarkers, including mannan antigen/anti-mannan antibody and 1,3-β-D-glucan (BDG), both components of the yeast cell wall, a nano-diagnostic method utilizing T2 magnetic resonance, and *Candida* DNA detection by PCR-based techniques have been investigated as adjuncts to body fluid cultures and have shown promising results in improving diagnostic efficacy and shortening detection time in neonatal populations. This review aims to provide an overview of the diagnostic tools and the current management strategies for invasive candidiasis in neonates. Timely and accurate diagnosis followed by targeted antifungal treatment can significantly improve the survival and outcome of neonates affected by *Candida* species.

## 1. Introduction

### 1.1. Epidemiology

Invasive candidiasis (IC) represents one of the leading causes of morbidity and mortality in neonatal intensive care units (NICUs) and is reported to be the third most common cause of late-onset neonatal sepsis [1,2]. The incidence of IC shows considerable variation across geographic areas and even between different centers in the same region [2,3,4,5,6,7,8]. Preterm and/or low birth weight neonates represent the most vulnerable population, and the prevalence of IC is inversely correlated with gestational age and birth weight [9]. The reported incidence among NICU admissions is estimated to be between 0.5 and 2%; however, among the extremely low birth weight (ELBW) neonates, the reported incidence rises up to 20% [1,2,9].

Invasive candidiasis in preterm neonates is associated with significant morbidity and mortality, reported up to 50% in ELBW neonates populations [10,11]. A composite outcome of death or neurodevelopmental impairment was observed in 73% of ELBW neonates with IC [12]. Moreover, a recent study demonstrated that 44% of neonates with IC exhibited adverse neurodevelopmental outcomes, a rate that was significantly higher than that observed in survivors of non-fungal infections [11].

### 1.2. Microbiology and Pathogenesis

*Candida* spp. represent a common constituent of the human normal flora with the capacity to manifest pathogenic behavior. The potential for *Candida* species to cause invasive infections has been associated with specific virulence factors, which may vary depending on the strain, the site of infection, and the host immune response. These factors include adherence and invasion of the host cells, formation of biofilms in tissues and indwelling devices, the transition from yeast to hyphae, and the production of tissue-damaging enzymes [13,14].

In neonatal invasive infections, *Candida albicans* is the most commonly identified strain, followed by *Candida parapsilosis*. Less frequently, *Candida glabrata*, *Candida tropicalis*, *Candida krusei*, and the recently emerging *Candida auris* are identified. [8,11,15,16,17]. However, species distribution varies in different geographical regions. A higher proportion of non-*albicans* species is observed in developing countries. It is worth noting that the susceptibility pattern of different strains of *Candida* to antifungal drugs varies, and, therefore, it is crucial to identify the causative strain [4,5,6,18,19,20].

Neonates in the NICU, especially premature and low birth weight neonates, represent a population with a high rate of *Candida* colonization. *Candida* spp. can be transmitted either vertically, during vaginal delivery from a colonized mother, or horizontally from the NICU environment [21,22,23,24]. It has been reported that almost 60% of very low birth weight (VLBW) neonates are colonized during the first weeks of their NICU stay, and about 20% of them will develop IC [21]. Colonization with *Candida* species represents the first step in the pathogenesis of systemic infections. Although colonization does not invariably lead to invasive disease, it may be followed by *Candida* translocation and dissemination in the presence of predisposing conditions [23].

### 1.3. Risk Factors

The risk of IC is inversely correlated with gestational age and birth weight [9]. This is attributed to the immature immune system and natural protection barriers of preterm neonates, as well as the need for prolonged NICU stay. Invasive procedures, including central venous catheters (CVC) and endotracheal tubes, disrupt epithelial barriers, thereby permitting the invasion of pathogens and subsequent dissemination [25,26,27].

Administration of broad-spectrum antibiotics, especially third-generation cephalosporins, and carbapenems, which are known to suppress the normal microbiota of the gastrointestinal tract, is a well-recognized predisposing factor for IC [28,29]. Corticosteroids, due to their immunosuppressive effects, and H2-antagonists, due to the alkalization of gastric pH, which modifies normal bacterial flora, have been proposed to promote microbial dysbiosis [30,31,32]. Furthermore, the delay in the achievement of full enteral feeding and parenteral nutrition administration, particularly lipid emulsion, is a well-established predisposing factor for *Candida* colonization and replication [33,34,35].

Gastrointestinal pathologies, such as prior abdominal surgeries and necrotizing enterocolitis (NEC), are known to predispose to IC due to the impairment of the intestinal barrier, which permits the translocation of *Candida* into the circulation [8,36,37].

It has been reported that colonization of more sites and increased colonization density represent risk factors for yeast translocation and dissemination, potentially leading to invasive disease [38].

Changes in NICU practice, including the avoidance of modifiable predisposing factors by reducing broad-spectrum antibiotic administration, accelerating enteral feeding advancement, and early removal of CVCs, along with the administration of prophylactic antifungals to high-risk neonates, have been demonstrated to be an efficient strategy for decreasing the incidence of IC.

### 1.4. Clinical Presentation

The clinical picture of neonates with IC is often not differentiated from that of a bacterial late-onset infection, as the symptoms are typically non-specific. Sepsis-like symptoms and signs, including apnea, respiratory distress, lethargy, temperature instability, feeding intolerance, and cardiovascular instability, may be presented [1,37,39].

Candidemia has the potential to disseminate in different organ systems through the blood or by the formation of septic emboli, which can result in deep-tissue infections and the development of fungal masses [1,40]. Dissemination in the central nervous system (CNS) is a relatively frequent sequela, manifesting as meningitis or encephalitis or less commonly as ventriculitis or brain abscesses [40,41]. The spectrum of renal involvement extends from cystitis to parenchymal infiltration, calyceal mycetoma, and the formation of fungal masses, which can result in obstructive uropathy [42,43,44]. Endocarditis is a rare but serious complication, often associated with long-lasting candidemia and the presence of a central venous catheter [45]. Less common complications of IC include eye involvement (chorioretinitis or endophthalmitis), osteoarticular infections (arthritis or osteomyelitis), liver and spleen abscesses, and embolic skin abscesses [46]. A potential involvement of *Candida* infections in the pathogenesis of spontaneous intestinal perforation (SIP) has been proposed [47].

In consideration of the potential involvement of different organ systems, neonates diagnosed with IC should undergo a comprehensive evaluation to accurately determine the extent of the disease. According to the Infectious Diseases Society of America (IDSA) guidelines, a lumbar puncture and cerebrospinal fluid culture (CSF) and fundoscopy should be practiced in all neonates with positive blood or urine cultures for *Candida* spp. Moreover, imaging of the genitourinary tract, liver, and spleen is recommended in cases of persistent candidemia, as evidenced by persistent *Candida* positivity [48].

The aim of this narrative review is to summarize the existing and emerging literature on the diagnosis and the management of invasive *Candida* infections in the neonatal intensive care unit (NICU). The PubMed and Google Scholar databases were searched for relevant studies up to August 2024 using the following terms: neonatal invasive candidiasis, preterm neonate, candidiasis diagnosis, candidiasis treatment, antifungal agents, amphotericin, fluconazole, and echinocandins. Ultimately, 176 articles were found, and 94 were included, particularly randomized control trials, systematic reviews, narrative reviews, and observational studies. Furthermore, the reference lists of the retrieved articles were reviewed to assess for the presence of relevant studies that may have not been detected in the initial search.

## 2. Diagnosis

An early and accurate diagnosis of systemic candidiasis, followed by the prompt administration of antifungal treatment, is crucial for survival and the elimination of long-term sequelae. However, the diagnosis is challenging due to the non-specific clinical presentation and, therefore, relies on diagnostic testing. While blood culture is considered the gold standard for IC diagnosis, this method has significant disadvantages, and alternative laboratory techniques have been investigated to facilitate a timely and precise diagnosis (Table 1).

### 2.1. Blood Culture

Blood culture is considered the gold standard for IC diagnosis in all age groups. However, considerable constraints exist, including the slow turnaround time and the limited diagnostic accuracy [70,74].

The sensitivity threshold for blood cultures is ≤1 colony-forming unit per milliliter (cfu/mL), with the detectability of *Candida* species contingent upon the volume of blood sampled [49,50,51]. Lancaster et al. employed in vitro techniques to investigate the minimum blood volume required for the isolation of *Candida* spp. from blood cultures exhibiting low and ultra-low concentrations. *Candida albicans* and *Candida parapsilosis* were recovered from blood specimens of 0.5 mL volume at a load of 1–10 cfu/mL. However, ultra-low concentrations (i.e., <1 cfu/mL) required a 3 mL blood volume for isolation [75]. In neonates, the detection of *Candida* is challenging due to the difficulty in obtaining adequate blood volumes [50]. According to the European Society of Clinical Microbiology and Infectious Diseases (ESCMID) recommendations, three blood culture specimens should be obtained in a single session with a total volume of 2–4 mL for neonates weighing less than 2 kg [76]. The IDSA and the American Society for Microbiology recommend a single culture of 2 mL for neonates < 1 kg and two cultures of 2 mL each for neonates weighing 1–2/kg [77]. Obtaining the recommended blood volume for culture in neonates is often unfeasible due to either hemodynamic instability or the difficulty of obtaining the sample. Harewood et al. observed that more than one-third of neonatal blood cultures contained negligible amounts of blood [78,79].

Even with a sufficient volume of blood cultures, the overall sensitivity in diagnosing IC is estimated to be below 50% [9,70,80]. A further limitation of blood cultures in the diagnosis of IC is the slow turnaround time, which typically ranges from 1 to 3 days [49,70]. A previous retrospective study demonstrated that in neonates diagnosed with IC, the median time to positive blood culture was 36 h if not on antifungal drugs and 42 h when antifungal therapy was initiated [81]. A delay in the initiation of therapy, pending culture results, has been associated with a worse clinical outcome [82]. Nevertheless, the *Candida* strain and the system employed influence the sensitivity rate and turnaround time [76,83]. The use of fungal selective media has been associated with enhanced sensitivity in a shorter time frame [84,85].

### 2.2. Serum Biomarkers

#### 2.2.1. Mannan/Anti-Mannan Antibody

Distinctive polysaccharides are present in fungal cell walls, and the detection of these antigens, such as mannan antigen for *Candida* spp. and galactomannan for *Aspergillus* spp., has been used as biomarkers for the diagnosis of fungal infections [70]. Mannan is a high-molecular-weight polysaccharide that constitutes a component of the upper layer of the *Candida* cell wall [74,86]. The detection of mannan antigen and anti-mannan antibody has been proposed as a diagnostic marker for IC, but limited data exist for use in the neonatal population [55]. The most widely used testing assay is the combined mannan/anti-mannan antibody assay, PLATELIA™ *Candida* Ag Plus system (Bio-Rad Laboratories, Marnes-la-Coquette, Paris, France) [74,87]. Olivieri et al. studied the efficacy of PLATELIA™ in the diagnosis of IC in a neonatal cohort and observed a sensitivity and specificity of above 94%. It is noteworthy that the test result was positive at a median of 8.5 days prior to the detection of positive cultures, which indicates the potential usefulness of this biomarker in the prompt diagnosis of IC in high-risk neonates [56]. In a prospective study, Montagna et al. reported the presence of positive mannan antigen in five out of seven neonates with IC. It is notable that in both neonates with IC and a negative mannan antigen result, *C. parapsilosis* was isolated [57]. The limited sensitivity of the mannan antigen in the detection of *C. parapsilosis* and *C. krusei* has been observed in several studies and is likely attributable to variations in mannose epitopes [56,74]. A recent prospective case-control study examined the mannan antigen in *Candida* colonized and non-colonized neonates and observed that the test results were not influenced by the presence of *Candida* colonization [55]. However, due to the accelerated elimination of the antigen from the circulation, repeated testing is necessary [57,84].

#### 2.2.2. 1,3-β-D-Glycan

The 1,3-β-D-glycan (BDG) is a component of the inner cell wall of a variety of pathogenic fungi, including *Candida* species. Elevated levels of BDG have been observed in patients with IC, and thus BDG has been proposed as a potential biomarker for early candidiasis diagnosis [59,60].

The Fungitell Assay (Associates of Cape Cod, Inc., East Falmouth, MA, USA) is the most widely used test for quantifying BDG [70,74]. Several studies have been conducted on neonatal populations, with the objective of investigating the utility of BDG as a biomarker for IC and the optimal cut-off levels for positivity. This method’s significant advantages include prompt results and the minimal quantity of blood required for the assay (<100 μL) [58]. As specified by the manufacturer of the Fungitell Assay, a positive result is indicated by a cut-off level of 80 pg/mL [61]. Nevertheless, a number of studies have argued that this threshold may not be appropriate for use in neonates and have proposed a higher threshold for IC diagnosis [61,69,88,89]. In the CANDINEO study, utilizing the aforementioned threshold in VLBW neonates, the positive predictive value was estimated to be 14%, while the negative predictive value was 97.1% [69]. Cliquennois et al., in a prospective cross-sectional study, reported a sensitivity and specificity of 85.7% and 51.9%, respectively, of BDG in the diagnosis of IC with a cut-off of 80 pg/mL and proposed that the optimal threshold could be 174 pg/mL [61]. According to the results of a recent review and meta-analysis, the sensitivity and specificity of the Fungitell Assay in the neonatal population at a threshold of 80 pg/mL were estimated at 89% and 60%, respectively, and at a cut-off of 120 pg/mL were 81% and 80%, respectively. The authors concluded that BDG could be useful in excluding IC and potentially as an adjunctive method in the diagnosis of neonatal IC; however, they acknowledged that data are scarce in the neonatal population [58].

A further aspect of BDG as a biomarker of systematic candidiasis is monitoring the response to antifungal treatment. A limited number of studies in the neonatal population have performed serial measurements of BDG levels to assess the response to therapy and have observed an initial increase and then a progressive decline of serum BDG levels [59,88,90].

One notable limitation of the BDG as a biomarker for IC is the high proportion of false-positive results. A number of potential contributors have been identified, including glycan-containing gauzes, hemodialysis membranes, and the administration of specific beta-lactam antibiotics, blood products, intravenous immunoglobulin, albumin, and postnatal corticosteroids. Moreover, it has been proposed that Gram-positive and Gram-negative sepsis and *Candida* colonization may be associated with elevated BDG levels [58,60,69,70,74,88]. It should be noted that BDG is a cellular component of many pathogenic fungi in addition to *Candida* spp., including *Aspergillus* spp., *Malassezia* spp., and a variety of others, and therefore the ability to specifically diagnose *Candida* infections is precluded [49,60,88,91].

### 2.3. Molecular Techniques

In systemic *Candida* infections, *Candida* species identification and antifungal susceptibility testing are critical for effective treatment. Different species exhibit varying degrees of inherited and acquired antifungal resistance, and prompt initiation of the appropriate antifungal agent significantly improves survival. The shift towards non-albicans *Candida* infections in recent years has complicated the choice of the empirical antifungal agent. In contrast to the generally susceptible *Candida albicans* strain, different non-*albicans* species demonstrate a range of resistance patterns to antifungal agents [92]. *Candida krusei* shows innate resistance to fluconazole; *Candida glabrata* is characterized by low azole susceptibility, and resistance is increasing worldwide, and fluconazole-resistant *Candida parapsilosis* has recently emerged globally [93,94,95]. In addition, *Candida auris*, a multi-drug-resistant strain, is rapidly emerging around the world [16].

Prompt species identification is therefore essential for the appropriate choice of antifungal agent in invasive candidiasis. To achieve this, advanced molecular techniques capable of rapid and accurate speciation of *Candida* have been developed, including spectroscopy-based methods, DNA-based techniques, and sequencing.

#### 2.3.1. MALDI-TOF/MS and PNA-FISH

Conventional blood culture speciation techniques are characterized by a prolonged turnaround time, typically between 24 and 72 h, and a relatively low level of precision [49,96]. To overcome the aforementioned shortcomings of conventional biochemical and phenotypic-based identification techniques, a number of newer, advanced molecular methodologies have been developed that can accurately identify *Candida* species from blood culture broths [49,92]. The matrix-aided laser desorption/ionization time-of-flight mass spectrometry (MALDI-TOF/MS) technique is based on sample ionization and the subsequent calculation of the mass-to-charge values of the ionized proteins. These values are then compared to reference values that have been standardized and entered into a database [97,98]. The technique has been demonstrated to be accurate and capable of discerning over 200 bacterial and fungal species, including rare *Candida* species, in less than 15–20 min [49,92,99]. Another useful molecular technique capable of accurately and rapidly identifying *Candida* species from positive blood culture broths is the peptide nucleic acid fluorescent in situ hybridization (PNA-FISH). This technique is based on the detection of hybridization of peptide nucleic acid probes specific for rRNA regions in *Candida* strains through the use of fluorescent microscopy. Nevertheless, the range of *Candida* species identified by this method is relatively narrow and contingent upon the specific test system employed [49,92,96]. However, a significant disadvantage of the two techniques mentioned above, MALDI-TOF/MS and PNA-FISH, is that the cost of the necessary equipment precludes their use in low-income settings [49].

#### 2.3.2. T2 Magnetic Resonance (T2MR) Assay

T2 magnetic resonance (T2MR, T2 Biosystems, Lexington, MA, USA) technology is an innovative molecular technique that utilizes magnetic resonance combined with nanotechnology to identify pathogens. The T2*Candida* system, an FDA-approved assay, can detect five *Candida* species (*Candida albicans*, *Candida tropicalis*, *Candida parapsilosis*, *Candida glabrata*, *and Candida krusei*) in whole blood specimens using T2MR technology [49,84]. The sensitivity threshold varies between *Candida* species and has been defined as 1 cfu/mL for *C. tropicalis* and *C. krusei*, 2 cfu/mL for *C. albicans* and *C. glabrata*, and 3 cfu/mL for *C. parapsilosis* [63,65].

In a retrospective study in a pediatric cohort, T2*Candida* showed 100% sensitivity and 94.1% specificity [60]. One of the most notable advantages of this assay is the rapid turnaround time and speciation, which facilitate the prompt and targeted administration of antifungal therapy [63]. In the aforementioned study, the mean time for *Candida* identification was 3.7 h using the T2*Candida* assay, significantly shorter than the mean time of 125.5 h for positive blood culture results [64]. Despite the lack of data on the utility of T2*Candida* as a diagnostic tool for IC in neonatal and pediatric populations, the available data are consistent with those of larger studies involving adults. A recent meta-analysis of eight studies on adult populations revealed a pooled sensitivity of 91%, a specificity of 94%, and a time to positivity of 3–4 h [100].

According to the manufacturer’s specifications, the quantity of blood required for the assay is 3 mL; however, in studies with pediatric populations, a reduced volume of blood has been employed, either by pipetting samples directly into the T2*Candida* cartridge or by diluting with General Purpose Buffer, without compromising the assay’s sensitivity [64,67].

According to the current literature, previous antifungal treatment does not impact the T2*Candida* assay’s results, in contrast to the effects observed in blood cultures. Therefore, T2*Candida* may represent a useful tool for monitoring the response to treatment in patients treated with antifungal agents [64,101].

#### 2.3.3. Polymerase Chain Reaction (PCR) Assays

A variety of PCR techniques have been investigated to facilitate the early and accurate diagnosis of IC, either by targeting specific strains of fungi or by detecting fungal DNA in general (“panfungal” PCR) [87,102]. Despite the availability of several commercial PCR assays, they are not FDA-approved for *Candida* infections, and their role in the diagnostic pathway of IC remains undefined, especially in neonatal populations in which data are very limited [69,102]. The principal benefits of PCR-based methodologies are the rapid turnaround time, providing accurate strain identification in 2 to 4 h, the increased sensitivity compared to blood cultures, the reduced blood volume necessary, the high negative predictive value (NPV) in low prevalence settings, and the ability to monitor the patient’s response to antifungals [49,50,69].

The primary challenges associated with PCR as an IC diagnostic test are its suboptimal standardization, the considerable range of sensitivity observed, and the necessity for DNA extraction and purification [102]. The extraction of fungal DNA represents a pivotal stage in the molecular diagnosis process. The efficacy of fungal cell lysis and the quality of the DNA recovered subsequently influence the sensitivity and specificity of the assay [103]. *Candida* cell lysis is challenging due to the consistency of the cell wall and requires high temperatures or toxic agents to achieve [92,104]. A variety of methods for DNA extraction have been utilized, including enzymatic, chemical, and mechanical techniques. However, the optimal method has yet to be identified [104,105,106]. Particularly for the neonatal population, the ideal method would require a minimal amount of blood to detect low fungal concentrations or techniques that are effective regardless of the origin of the biological sample, thus increasing the potential for detection of the pathogen [104].

A significant limitation of PCR techniques is the potential for amplification of contaminating traces of fungal DNA, leading to false-positive results. This is more commonly observed with assays targeting a broad range of pathogens, such as pan-fungal PCR, rather than assays targeting specific species, and when the most sensitive assays are employed due to testing of samples with low fungal load as peripheral blood [107,108]. The potential for contamination arises from a number of sources, including airborne environmental pathogens, contaminated surfaces, improper handling, contamination during the process of DNA extraction, and reagents or consumables. A variety of decontamination techniques have been employed at each stage of the specimen processing procedure in order to mitigate this potential issue and to prevent the obtainment of misleading results [109]. The selection of decontamination techniques should be made with the objective of effectively eliminating any contaminating material, while ensuring that the efficacy and sensitivity of the procedure are not compromised [110]. All handling procedures should be conducted in laminar air flow (LAF) benches equipped with ultraviolet (UV) light for decontamination purposes. Furthermore, hypochlorite solution should be utilized for the cleaning of all equipment and consumables [108,110]. DNA-free reagents and consumables should be used when feasible [107]. However, if decontamination of reagents is required, the choice of decontamination technique should be based on the characteristics of the specific reagent. Methods using UV light, γ-irradiation, and various enzymes such as double-strand specific DNase (dsDNase) have been used and shown to be effective [110].

In the CANDINEO study, a multicenter study involving VLBW, the sensitivity and specificity of PCR in diagnosing IC were reported to be 87.5% and 81.6%, respectively. The reported NPV of the PCR assay was 98.8%, underscoring the potential clinical utility of these techniques in the cessation of unnecessary antifungal treatment. Moreover, in 17.4% of cases, PCR was positive despite the negative blood cultures [69]. Furthermore, the enhanced diagnostic yield of PCR was demonstrated in another study conducted on a pediatric population, in which PCR was positive in 24% of cases suspected of candidemia and blood cultures in 14.8% [111]. The limited data available regarding the PCR in neonates and children are consistent with the evidence from studies conducted in adults. In a meta-analysis, Avni et al. reported a PCR sensitivity and specificity of 93% and 95%, respectively, and positivity rates of PCR of 85% in patients with proven or probable systemic candidiasis, compared to 38% of positive blood culture results [112].

The novel Droplet Digital PCR (ddPCR) technology, which involves randomly encapsulating pathogen nucleic acid in microdroplets and a separate reaction in each one, offers several advantages, including the ability to detect pathogens rapidly, even in minute quantities, and to quantify the target genetic material with great precision in biological samples [113]. The utility of ddPCR has been investigated in a neonatal population, with reported sensitivity and specificity of 86% and 100%, respectively, and a detection limit of 3.2 copies/μL [114].

The cationic conjugated polymer-based fluorescence resonance energy transfer (CCP-FRET) technology has recently been developed as an innovative method for diagnosing IC. The two components of the CCP-FRET assay are a water-soluble conjugated polymer and a fluorescence dye-labeled pathogen-specific DNA. This technique is rapid, providing pathogen identification within three hours, with a detection limit as low as one-tenth that of real-time PCR. It has been demonstrated to have a sensitivity and specificity of up to 100% in clinical specimens. Moreover, the assay necessitates a minimal blood volume of 0.2 mL, which is of particular significance in neonates. It is important to note that the selection of appropriate primers is essential for the efficacy of the assay. However, more research is required to optimize the technique [70,115].

#### 2.3.4. Next Generation Sequencing (NGS)

Metagenomics (mGNS) is the application of NGS to detect the genomic material of a number of microorganisms simultaneously in various biological specimens [102]. In a retrospective study of children with hematological diseases and probable sepsis, the rate of positive results using mNGS was 57.2%, significantly exceeding that of blood cultures (12.5%) [116]. A recent meta-analysis of studies conducted in neonatal and pediatric populations concluded that mGNS could be a valuable tool for identifying pathogens in cases of sepsis, offering a particular advantage in cases where the causative pathogen is an unusual or difficult-to-isolate organism, such as fungi [71]. Despite the indisputable advantages of metagenomics as a diagnostic method for the identification of fungi, bacteria, viruses, and mixed infections from a single specimen, this method presents several noteworthy limitations. These include the incapacity to distinguish between colonization and infection, the lengthy turnaround time, and the high cost [72,74].

### 2.4. Candida Diagnosis in Middle-Low Income Countries

Although significant progress has been made in *Candida* diagnostics with the development of techniques offering rapid and accurate *Candida* identification, availability is not widespread beyond developed countries, as the high cost of these techniques precludes their use in middle and low-income countries [117].

The high cost of the more sensitive automated culture systems renders them inaccessible in low-income settings, and manual systems are more widely used. Conventional phenotypic and biochemical assays are more commonly employed. However, these assays are known to have a slow turnaround time and limited sensitivity and specificity [117,118]. Furthermore, the restricted capacity of conventional techniques to distinguish between species is of particular significance, given the increased prevalence of non-*albicans Candida*, such as the recently emerged *Candida auris*, in these settings. Accurate identification is crucial for the effective initiation of antifungal therapy [16].

Among serum biomarkers, mannan and anti-mannan antibodies are cost-effective and employed in numerous centers in developing countries [118]. Implementing advanced molecular techniques is unfeasible in low-income regions due to the high cost of the requisite equipment. However, isothermal techniques and conventional PCR assays may be employed in settings where resources are limited [117,119].

### 2.5. Candida Auris Diagnosis

The diagnosis of *Candida auris* is a considerable challenge. Conventional fungal identification techniques based on phenotypic and biochemical characteristics are unreliable for the diagnosis of *C. auris*, as differentiation from other uncommon *Candida* spp., including *C. haemulonii*, *C. famata*, and *Sacharomyces* spp., is often not feasible. [16,120,121]. More accurate diagnosis is possible with newer techniques, such as MALDI-TOF MS [122]. Furthermore, a number of molecular methods have been developed for the prompt detection of *C. auris*, including PCR assays, T2MR, and loop-mediated isothermal amplification (LAMP), but these are not always readily available in all settings [16]. Due to the complexity of diagnosis and limited resources in most laboratories, *Candida auris* is probably underdiagnosed, and prevalence cannot be estimated with certainty [16].

### 2.6. Challenges of Candida Diagnosis in Neonates

Diagnosis of neonatal invasive candidiasis is challenging. The clinical presentation is non-specific, and the diagnosis relies on laboratory techniques. Clinicians should aim to have an accurate diagnosis while limiting the quantity of blood obtained. However, this poses a significant challenge. The sensitivity of blood culture depends on the volume of blood obtained and is reduced with the usual volume obtained in neonates. In addition to blood cultures, other techniques, including T2MR*Candida* and specific DNA extraction methods, may require blood volumes that are infeasible to obtain in a low-birth-weight or hemodynamically unstable neonate. A further limitation in the diagnosis of *Candida* in the neonatal population is that the majority of diagnostic techniques have been validated in adult or pediatric patients, and data in neonates is limited, such as for the Fungitell Assay, for which the cut-off in neonates has not been determined. It is important to recognize the limitations of diagnostic tests in this vulnerable population and to carefully select the most appropriate and accurate diagnostic tests.

## 3. Treatment

The timely initiation of antifungal treatment in neonates with disseminated candidiasis has been demonstrated to have a critical impact on survival rate. The efficacy and safety of agents from four classes of antifungals have been evaluated in infants and neonates: polyenes, triazoles, echinocandins, and nucleoside analogues. The various classes of antifungal drugs act via disparate mechanisms, including the disruption of cell membrane biosynthesis, cell wall synthesis and stability, and fungal DNA/RNA synthesis [123,124].

### 3.1. Antifungal Agents

#### 3.1.1. Polyenes

Polyene macrolides are the oldest category of antifungal drugs. Amphotericin B deoxycholate (D-Amb) represents one of the first-choice agents for neonatal systemic *Candida* infections [123,125] (Table 2). Amphotericin B acts by binding to ergosterol, a component of the yeast cytoplasmic membrane, leading to pore formation, increased permeability to electrolytes, and, ultimately, cell death [126]. Nevertheless, D-Amp has the potential to bind to cholesterol within the membranes of mammalian cells, which is postulated to be the causative factor of the observed side effects, including nephrotoxicity [127].

To decrease the incidence of side effects, new formulations of the drug combined with lipids were developed [125]. However, in most settings, D-Amp is preferred over lipid formulations in neonatal systemic candidiasis [48,128]. A multicenter observational study reported a significantly higher mortality rate in neonates with IC treated with liposomal amphotericin B (L-Amb) than with D-Amb. The authors hypothesize that this is probably attributable to the poorer penetration of L-Amb to the kidneys or inappropriate dosing in neonates [129]. In comparison to D-Amp, L-Amp demonstrates a restricted capacity to penetrate the urinary tract, which is frequently implicated in neonatal systemic candidiasis [48]. However, in a prospective historical control multicenter study in a VLBW population, the two formulations of amphotericin showed comparable efficacy [130].

D-Amb lacks enteral absorption and is administered intravenously [123,125]. The faster elimination observed in neonates is presumably the reason for the reduced nephrotoxicity compared to older children and adults [123]. Le et al. reported an incidence of nephrotoxicity of 16% in neonates treated with D-Amp. In the majority of cases, the nephrotoxic effects were transient [129]. Consistent with the observations of previous studies, Ambreen et al. noted that maintaining adequate hydration and sodium intake above 4 mEq/kg/day throughout the course of D-Amb therapy exerts a protective effect with regard to the development of nephrotoxicity in neonates [131]. In addition to nephrotoxicity, hypokalemia, infusion-related reactions, and hepatotoxicity are reported side effects of amphotericin, although these appear to affect neonates less frequently than older patients [132].

Amphotericin has been reported to penetrate CSF well in neonates. Although studies in adults report CSF levels as low as 2–4% of serum concentration, Bailey et al. detected CSF levels of amphotericin B in preterm neonates at 40–90% of serum values. In a rabbit model of *Candida* meningoencephalitis, D-Amb and L-Amb exhibited superior antifungal efficacy relative to alternative amphotericin formulations. However, higher concentrations of L-Amb were achieved in brain tissue compared to those of D-Amb [133].

**Table 2:** Infectious Diseases Society of America (IDSA) and European Society of Clinical Microbiology and Infectious Diseases (ESCMID) guidelines for the management of neonatal invasive candidiasis.

	IDSA (2016) [48]		ESCMID (2012) [134]
	*Candida* Bloodstream Infection	*Candida* CNS Infection	
Antifungal agent Agents of choice	D-Amb 1 mg/kg/day or fluconazole 12 mg/kg/day if not on fluconazole prophylaxis	D-Amb 1 mg/kg/day	D-Amb 1 mg/kg/day or L-Amb 2.5–7 mg/kg/day or fluconazole 12 mg/kg/day if not on fluconazole prophylaxis (loading dose 25 mg/kg/day can be considered)
Alternatives	L-Amb 3–5 mg/kg/day as an alternative (caution if urinary tract involvement)	L-Amb 5 mg/kg/day as an alternative	ABLC 2.5–5 mg/ kg/day as an alternative
	Echinocandins with caution, as salvage therapy or when D-Amb or fluconazole cannot be used due to toxicity or resistance	Flucytosine, 25 mg/kg four times daily, may be added in patients who do not respond clinically to initial AmB therapy	Micafungin 4–10 mg/kg/day
		After response to initial treatment, step down to fluconazole 12 mg/kg daily for susceptible isolates	Capsofungin 25 mg/m^2^/day (limited data available)
Implanted devices	CVC removal is strongly recommended	It is recommended that CNS devices should be removed if possible	Removal or replacement of intravenous catheters and/or other implanted prosthetic devices should be considered
Therapy duration	2 weeks after blood culture sterilization and resolution of signs of candidemia	Continue therapy until all signs, symptoms, and CSF and radiological abnormalities have resolved	2 weeks after blood culture sterilization provided that no unresolved deep infection remains

D-Amb: amphotericin B deoxycholate; L-Amb: liposomal amphotericin B; ABLC: amphotericin B lipid-complex; CVC: central venous catheter; CNS: central nervous system; CSF: cerebrospinal fluid.

#### 3.1.2. Triazoles

Triazoles represent a major class of antifungal drugs widely used in the neonatal population. The antifungal activity of triazoles is achieved through the disruption of ergosterol biosynthesis, a crucial constituent of the fungal cell membrane. This is achieved by the inhibition of 14-a-sterol demethylase, a cytochrome P-450 enzyme [124,125].

Among triazoles, fluconazole, a first-class triazole, is the most thoroughly studied and widely used agent for the prophylaxis and treatment of IC in neonates. Fluconazole has been demonstrated to be effective against the majority of *Candida* species; however, resistance has been documented in *Candida glabrata* and *Candida krusei* [124]. Fluconazole demonstrates excellent penetration into the CNS and vitreous body [125]. A significant benefit is the high oral bioavailability (>90%) of the drug [135,136]. The most frequent side effects of fluconazole in neonates are gastrointestinal irritation and hepatotoxicity [135,137]. As fluconazole is renally excreted, dose modification is required in patients with renal impairment [123,135]. The inhibition of cytochrome P450 enzymes by azoles may result in interactions with other pharmaceutical agents, potentially affecting the therapeutic efficacy [125]. The literature on the comparative efficacy of fluconazole and amphotericin B in neonatal candidiasis is limited, resulting in a lack of consensus regarding the optimal first-line agent in NICUs [138,139,140]. However, fluconazole, in addition to its high oral bioavailability, has the advantage of being compatible with drugs commonly used in the NICU, as opposed to amphotericin, when administered intravenously [135].

Itraconazole, another first-generation triazole that demonstrates fungistatic and fungicidal activity, is generally well tolerated in pediatric patients and available in an oral formulation [137]. However, oral bioavailability varies and is dependent on gastric pH and food intake [74]. Mondal et al., in an RCT involving 43 pediatric patients with systemic candidiasis, reported comparable efficacy and safety of itraconazole and fluconazole [141]. Moreover, a systematic review of 32 studies concerning systematic fungal infections in infants revealed a similar conclusion regarding the use of itraconazole [142]. However, the use of itraconazole in neonates is limited due to its highly variable pharmacokinetics, the lack of sufficient data on neonates, and the availability of alternative agents that have been subjected to more extensive investigation [137].

Voriconazole, a second-generation triazole, is a synthetic derivative of fluconazole with a broader spectrum of activity among *Candida* species, including *Candida glabrata* and *Candida krusei* [94]. It has also been demonstrated to exhibit efficacy against *Candida auris* [143]. Voriconazole demonstrates about 90% oral bioavailability, is characterized by moderate protein bound, and distributes well into tissues, including the CNS [123,144]. Adverse effects of voriconazole include hepatotoxicity, photosensitivity, and visual disturbances, which are reported to be transient in adults. Given the lack of safety data in the neonatal population, voriconazole is not recommended and should be used only in refractory cases as second-line therapy. However, it is not approved for use in children younger than 2 years [123,124].

#### 3.1.3. Echinocandins

Echinocandins are more recently developed antifungal drugs. They exhibit their fungicidal action by inhibiting the 1,3-b-glycan synthase complex, which leads to disruption of cell wall stability and, ultimately, lysis. As the target enzyme is absent in mammalian cells, echinocandins are generally well tolerated [124,125]. The results of a meta-analysis indicate that the prevalence of side effects necessitating treatment discontinuation was lesser in pediatric patients receiving echinocandins than in those treated with amphotericin B [145]. Echinocandins show poor oral bioavailability and are administered parenterally [123]. These agents have been demonstrated to exhibit a broad spectrum of activity against *Candida* species that are resistant to other antifungal agents and have also been proven to be efficacious in the eradication of *Candida* biofilms [146]. Echinocandins are characterized by a wide distribution to tissues, with the exception of the CNS and kidneys. In neonates, these sites are often affected in disseminated candidiasis, and high doses of echinocandins may be required to achieve optimal efficacy [74,124]. Micafungin is the only echinocandin approved for infant use [125].

A limited number of studies have been conducted to examine the efficacy, optimal dosing, and safety of micafungin in the neonatal population. Two RCTs involving neonates have documented that micafungin exhibits comparable efficacy to D-Amb and L-Amb in the treatment of systemic *Candida* infections [147,148]. Pharmacokinetic studies have demonstrated that micafungin has dose-dependent CNS penetration and that higher doses achieve CNS Candida eradication in neonates and young infants [149,150]. Although the urine excretion of active micafungin has been reported to be 0.7%, it has been postulated that the high plasma concentrations that are achieved may yield sufficient elevated levels in the urine to eradicate *Candida* from the urinary tract [151,152]. It is noteworthy that micafungin has been demonstrated to exert significant inhibitory activity against the adhesion and biofilm formation of various *Candida* species [153].

Micafungin is generally well tolerated, with a minimal propensity for drug-to-drug interactions [150]. The most commonly reported adverse reactions are gastrointestinal disturbances, hepatotoxicity, and hypokalemia [154]. The European Medicines Agency (EMA) has issued a “black box” warning due to the reported increased incidence of hepatocellular tumors in experimental animals after prolonged administration [134]. Nevertheless, a systematic review of nine studies reported a 73% efficacy of micafungin in infants with systemic candidiasis and an acceptable safety profile for both term and preterm neonates [155].

Caspofungin, another echinocandin, is not FDA-approved for infants younger than three months. Data on the use of caspofungin in neonates are limited, but the available literature suggests that the drug is both safe and effective [145,156,157,158].

Anidulafungin, a semi-synthetic lipopeptide, has the unique property that it is not metabolized but undergoes a process of slow degradation and biotransformation [159,160]. Anidulafungin is not currently licensed for neonatal use. The high doses that need to be administered to attain therapeutic CNS levels are associated with polysorbate 80 (PS80) accumulation [161]. However, in a recent prospective multicenter study, no PS80 accumulation was detected in pediatric patients aged > 1 month who received anidulafungin [162].

#### 3.1.4. Nucleoside Analogues

Flucytosine, a synthetic fluorinated analogue of cytosine, exerts its antifungal activity by disrupting RNA and inhibiting DNA synthesis in the fungal cell [159,163]. It is characterized by low protein binding, high hydrophilicity, and a wide distribution, including the CNS, the vitreous body, and urine [112,152]. Flucytosine is primarily excreted by the kidneys, and the elimination rate is proportional to the renal function. Thus, dose adjustments are necessary in cases of renal impairment, and caution is needed when administering the drug to premature neonates due to their immature renal function [64,152]. Another concern with the use of flucytosine is dose-related toxicity, including hepatotoxicity, bone marrow suppression, and gastrointestinal disorders [163]. Flucytosine monotherapy is not advised, due to the rapid development of resistance [71].

### 3.2. Central Venous Catheters (CVC)

CVCs are a common practice in the care of preterm and low-birth-weight neonates during their stay in the NICU, primarily for the administration of parenteral nutrition and intravenous drugs. It is well documented that systemic *Candida* infections are frequently associated with the development of biofilms on implanted medical devices [13,164,165]. Biofilms are attachment complexes composed of microbial cells integrated within an extracellular polymeric matrix composed of water, polysaccharides, proteins, lipids, and extracellular DNA [166,167]. The successful eradication of *Candida* biofilms presents a considerable therapeutic challenge, given that these structures provide protection for the fungus from antifungal drugs and the patient’s immune response. Consequently, biofilms act as reservoirs for the systemic dissemination and end-organ dissemination of pathogens, thus prolonging the infection [164,165].

According to the current guidelines, prompt removal of CVC in cases of neonatal systemic candidiasis is strongly recommended [48]. Benjamin et al. observed that prompt removal of CVC was associated with a shorter duration of candidemia and improved survival and neurodevelopmental outcomes in ELBW neonates [12]. In a recent study, Chen et al. identified delayed CVC removal as an independent risk factor associated with mortality in neonates with IC [168].

However, CVC removal is not always feasible, and the decision to proceed with removal should be made considering the necessity of maintaining central venous access in critically ill neonates [168,169]. In cases where maintaining central venous access is essential, lock therapy, i.e., instilling high concentrations of antifungals into the catheter lumen, has been proposed, but data on neonates are limited, and efficacy and safety have not been established [125]. The efficacy of various antifungal agents and combinations has been studied for lock therapy, including caspofungin, micafungin, anidulafungin, and L-AmB [170]. Ethanol-based solutions have also been shown to be highly effective and are a reasonable alternative [171].

### 3.3. Candida Auris Treatment

In recent years, there has been increasing global concern about the spread of *Candida auris*. The most concerning aspect of this *Candida* strain, which is capable of rapid dissemination in ICUs, is its multi-drug resistance profile [172,173]. *Candida auris* has been documented in the majority of cases to be resistant to fluconazole, the most commonly used antifungal agent for prophylaxis and treatment in NICUs. Resistance to fluconazole and amphotericin was reported in 97.4% and 67.1% of neonates, respectively, in a recent systematic review [16]. *Candida auris* is generally susceptible to echinocandins, although sporadic resistance has been reported, and the Centers for Disease Control and Prevention (CDC) recommends echinocandins beyond the neonatal period [16,172,173]. For neonatal *C.auris* infections, D-Amb is recommended as the first-line agent, followed by L-Amb in unresponsive cases. Micafungin or capsofungin are only recommended in exceptional cases without CNS involvement [174].

### 3.4. Antifungal Stewardship Programs

Establishing antifungal stewardship programs (AFS) is a crucial step in addressing the risks associated with the irrational use of antifungal agents. These risks have been well documented and include potential toxicities, drug-to-drug interactions, and the emergence of resistance [175].

Antifungal prophylaxis represents the most common indication for antifungal prescription in the NICU [176]. Fluconazole is the most frequently employed pharmaceutical agent for this purpose [176,177]. A recent multicenter study conducted in 12 NICUs in England reported that up to 80% of antifungal prescriptions in NICUs were given for prophylactic purposes, with less than 35% of these neonates having a clear indication according to guidelines [177]. The objective of the implementation of AFS is to facilitate the judicious use of prophylaxis when it is indicated in accordance with current guidelines and to ensure the selection of the most appropriate antifungal agent based on regional susceptibility patterns [175,177].

Empiric antifungal therapy is frequently initiated in preterm neonates due to the non-specific manifestations of systemic infection, the unavailability of rapid diagnostic testing in most settings, and the detrimental potential consequences of untreated disease. In the aforementioned study, 23% of neonates treated for IC had ultimately proven infection [177]. A treatment approach based on rapid diagnostics and the establishment of a susceptibility profile represents an essential component of AFS. It offers the opportunity to restrict the initiation of empirical antifungal treatment and direct therapy towards an efficacious agent and the optimal duration [175].

## 4. Conclusions

The diagnosis and management of invasive *Candida* infections remain a significant challenge in the NICU. Since blood culture, the long-standing gold standard for IC diagnosis, has severe limitations, biomarkers and innovative molecular diagnostic methods have been investigated, but the implementation of these techniques in routine clinical practice remains a future prospect. Accurate and early diagnosis is the key to effective and timely treatment, which improves outcomes, particularly for preterm neonates, who are at particular risk of mortality and long-term sequelae. A limited number of antifungal drugs have been approved for use in neonates, and future studies evaluating drugs currently used in adults and recently developed drugs will provide more opportunities for effective treatment.

## Figures and Tables

**Table 1:** Advantages and disadvantages of laboratory techniques and biomarkers for the diagnosis of neonatal candidiasis.

	Advantages	Disadvantages	Cost
Blood culture [49,50,51,52,53,54]	Antifungal susceptibility testing Sensitivity threshold up to <1 cfu/mL, depending on the blood volume	Sensitivity ~50% Challenging to obtain optimal blood volumes in neonates Slow turnaround time (1–3 days)	Low cost (estimated ~$10–30 per test), but highly variable depending on the culture system used
Mannan/anti-mannan antibody [55,56,57]	Early positivity High sensitivity and positivity (94.4%, 94.2%, respectively) High NPV	Low sensitivity for *C.parapsilosis*, *C.krusei* infections Fast elimination and repeat testing may be needed	Affordable test, specific cost varies depending on test used (estimated ~$20–30 per test)
1,3-β-D glucan [58,59,60,61,62]	Minimal amount of blood required (<100 μL) High sensitivity (>80%) High NPV Useful in treatment monitoring	The optimal positivity threshold in neonates is not yet determined Component of the cell wall of many fungal species, not specific for *Candida* spp. diagnosis Frequent false positive results	Affordable test (estimated ~$20–30 per test); high-cost equipment is necessary
T2MR assay [63,64,65,66,67]	High sensitivity and specificity Sensitivity threshold 1–3 cfu/mL, depending on species Rapid turnaround time Useful in treatment monitoring	Detection of five *Candida* species High blood volume required	High-cost equipment is necessary, estimated cost per test ~$150–265
PCR techniques [49,50,55,68,69,70]	High sensitivity and specificity High NPV Minimal blood volume required	Limited data on neonates Technique optimization needed	Cost highly variable depending on the assay (estimated from $10 to more than $100 per test), but high-cost equipment is necessary
NGS [71,72,73]	Detection of multiple microorganisms simultaneously	Inability to differentiate between colonization and infection Slow turnaround time	Requires highly expensive equipment, cost per test depends on sequencer used (estimated from $100 to more than $500 per test)

NPV: negative predictive value; T2MR: T2 magnetic resonance; PCR: polymerase chain reaction; NGS: next generation sequencing.

## Data Availability

Not applicable.
